# A new fibrin sealant as a three-dimensional scaffold candidate for mesenchymal stem cells

**DOI:** 10.1186/scrt467

**Published:** 2014-06-10

**Authors:** Vinícius P O Gasparotto, Fernanda C Landim-Alvarenga, Alexandre L R Oliveira, Gustavo Ferreira Simões, João F Lima-Neto, Benedito Barraviera, Rui S Ferreira

**Affiliations:** 1 Department of Tropical Diseases and Image Diagnosis. Botucatu Medical School, São Paulo State University (UNESP – Univ. Estadual Paulista), Av. Prof. Montenegro, Distrito de Rubião Junior, s/n., CEP 18618-970 Botucatu, São Paulo, Brazil; 2 Center for the Study of Venoms and Venomous Animals of UNESP (Centro de Estudos de Venenos e Animais Peçonhentos da UNESP) – CEVAP, São Paulo State University (Universidade Estadual Paulista) – UNESP, Rua José Barbosa de Barros 1780, Fazenda Experimental Lageado, CEP 18610-307 Botucatu, São Paulo, Brazil; 3 Department of Animal Reproduction and Veterinary Radiology, School of Veterinary Medicine and Animal Science, São Paulo State University (UNESP – Univ. Estadual Paulista), Av. Prof. Montenegro, Distrito de Rubião Junior, s/n., CEP 18618-970 Botucatu, São Paulo, Brazil; 4 Laboratory of Nerve Regeneration, Department of Structural and Functional Biology, University of Campinas – UNICAMP, Anatomy, Av. Bertrand Russel, s/n., Cx Postal 6109, CEP 13083-865 Campinas, São Paulo, Brazil

## Abstract

**Introduction:**

The optimization of an organic scaffold for specific types of applications and cells is vital to successful tissue engineering. In this study, we investigated the effects of a new fibrin sealant derived from snake venom as a scaffold for mesenchymal stem cells, to demonstrate the ability of cells to affect and detect the biological microenvironment.

**Methods:**

The characterization of CD34, CD44 and CD90 expression on mesenchymal stem cells was performed by flow cytometry. *In vitro* growth and cell viability were evaluated by light and electron microscopy. Differentiation into osteogenic, adipogenic and chondrogenic lineages was induced.

**Results:**

The fibrin sealant did not affect cell adhesion, proliferation or differentiation and allowed the adherence and growth of mesenchymal stem cells on its surface. Hoechst 33342 and propidium iodide staining demonstrated the viability of mesenchymal stem cells in contact with the fibrin sealant and the ability of the biomaterial to maintain cell survival.

**Conclusions:**

The new fibrin sealant is a three-dimensional scaffolding candidate that is capable of maintaining cell survival without interfering with differentiation, and might also be useful in drug delivery. Fibrin sealant has a low production cost, does not transmit infectious diseases from human blood and has properties of a suitable scaffold for stem cells because it permits the preparation of differentiated scaffolds that are suitable for every need.

## Introduction

Successful cell therapy requires the identification of appropriate cells for the intended purpose [1]. Mesenchymal stem cells (MSCs) are the most commonly used cells in tissue engineering [2-4] and are found in several organic compartments, including the bone marrow, blood vessels, skin, and fat and muscle tissues [5]. MSCs can differentiate into osteogenic cells [6-8], chondrogenic cells [8,9], adipogenic cells [10] and cardiogenic cells [11] in response to different stimuli.

MSCs are distinguished by their phenotype: MSCs express the cell surface markers CD73, CD90 and CD105 and are negative for CD11b, CD14, CD34, CD45 and human leukocyte antigen-DR [8].

The main strategy to deliver stem cells or growth factors to chronic wounds involves direct injection to the injury site. However, this method leads to poor engraftment and poor efficacy due to proteolytic degradation within the first few hours post injection, thus preventing the beneficial effects of these expensive biological agents [12]. In the field of tissue engineering, ideal scaffolds that permit cells to proliferate and differentiate without interference have been sought recently [3]. The main features of such scaffolds include the promotion of cell adhesion, mechanical support [13] and biodegradability [3].

Fibrin sealants have various applications in tissue engineering and could act as drug delivery vehicles and scaffolding matrices in surgical procedures [14] and might participate in inflammatory infiltrate chemotaxis during lesion repair [15-17]. According to Buchta and colleagues [18], sealants can improve and accelerate wound-healing processes, reduce blood loss and protect against bacterial infections. Additionally, the two components of fibrin sealants can be mixed and processed in different ways [19].

Bensaid and colleagues showed that MSCs that were seeded into fibrin scaffolds and subsequently grafted into mice could create extensive networks of blood vessels and fibroblasts, a result that was not observed when the sealant alone was grafted [20].

Fibrin sealant (FS), an innovative product derived from snake venom, is produced only from animal components and thus does not transmit human blood-borne infectious diseases. Moreover, the production costs are lower than those of commercial sealants [16]. This study thus evaluated the first use of FS as a cellular scaffold and demonstrated the effects of this biomaterial on the maintenance, induction, differentiation, integrity and viability of MSCs.

## Methods

### Fibrin sealant scaffold

Commercially available fibrin sealants consist of human thrombin and fibrinogen, which might transmit infectious diseases [16]. Researchers at the Center for the Study of Venoms and Venomous Animals at UNESP (Centro de Estudos de Venenos e Animais Peçonhentos da UNESP), São Paulo State University therefore proposed a new sealant produced from the thrombin-like enzyme extracted from snake venom and animal fibrinogen. The sealant was produced in this study according to the proposed standardization [15-17,21]. The product was provided in three microtubes that were stored at -20°C. Upon use, the components were mixed in the previously set proportions to generate a stable clot with a dense fibrin network.

### Mesenchymal stem cell expansion and characterization

Wistar rat MSCs were cultured in Dulbecco’s modified Eagle medium (high glucose; Gibco Laboratories, Grand Island, NY, USA) supplemented with 20% fetal bovine serum (Sigma-Aldrich, St Louis, MO, USA), 100 UI/ml penicillin, 100 μg/ml streptomycin solution (Gibco Laboratories) and 3 μg/ml amphotericin B (Gibco Laboratories) [22,23]. The experiments were performed according to the Brazilian College for Animal Experimentation (COBEA) guidelines and were approved by the University’s Ethics Committee in Experimental Animal Use (CEEA/FMB/UNESP, protocol number 811/2010).

First-passage MSCs were characterized by surface markers to determine the ratio between the MSCs and the hematopoietic stem cells in the culture. The results were analyzed with a flow cytometer (FACSCalibur; BD, San Jose, CA, USA) that was located at the Blood Center of the School of Medicine Botucatu (Hemocentro da Faculdade de Medicina de Botucatu). CD44 (HCAM OX50 FITC; Santa Cruz Biotechnology, Delaware Avenue, CA, USA) and CD90 (mouse anti-rat FITC; Gibco Laboratories) antibodies were used as positive markers for MSCs and a CD34 antibody (mouse anti-human FITC; BD Pharmingen, San Diego, CA, USA) was used as a negative marker.

The cell suspensions were adjusted so that each test had 1 × 10^5^ cells. The cells were distributed into five tubes (Falcon; BD Pharmingen) and were stained as follows: unstained cells (control 1), cells with only secondary antibody (control 2) and cells with either CD44, CD90 or CD34 antibodies.

The antibodies of CD44, CD90 and CD34 were added at a dilution of 1:10 for 30 minutes at room temperature. After antibody addition, 1 ml phosphate-buffered saline (PBS) was added to wash the cells and they were centrifuged at 2,000 rpm (620 × *g*) for 10 minutes. The supernatant was discarded and the pellet resuspended in 1 ml PBS. The CD44 marker was not conjugated and a secondary antibody was then added, waiting for 30 minutes before the supernatant was discarded and the pellet resuspended in 1 ml PBS. Finally the tubes were read on a flow cytometer (FACSCalibur; BD).

### Cell differentiation

The ability of the MSCs to differentiate into osteogenic, chondrogenic and adipogenic tissues was tested. The StemPro Adipogenesis Differentiation Kit, the StemPro Chondrogenesis Differentiation Kit and the StemPro Osteogenesis Differentiation Kit (all Invitrogen Life Science Technologies, Carlsbad, CA, USA) were used to differentiate MSCs into adipogenic, chondrogenic and osteogenic tissues, respectively. The kits were used according to the manufacturer’s instructions.

### Differentiation into the adipogenic lineage

When the cell culture reached 70% confluence, the complete culture medium was replaced with a specific differentiation medium that contained 90% StemPro Adipocyte Differentiation Basal Medium (Invitrogen Life Science Technologies), 10% StemPro Adipogenesis Supplement (Invitrogen Life Science Technologies), 100 UI/ml penicillin, 100 μg/ml streptomycin and 3 μg/ml amphotericin B (Invitrogen Life Science Technologies). The differentiation medium was replaced every 3 days during a 7-day period of differentiation. After this procedure, the cells were fixed in 4% paraformaldehyde, washed in PBS and distilled water and then stained with 2 ml Oil Red-O solution (Invitrogen Life Science Technologies, Carlsbad, CA, USA). The cells were incubated for 40 minutes at room temperature, after which the dye was subsequently removed and the cells were washed three times in distilled water. Two milliliters of hematoxylin solution were added to the cells for 15 minutes to label the nuclei. The wells were washed again with distilled water and the fields were observed under an inverted light microscope.

### Differentiation into the chondrogenic lineage

When the cell culture reached 70% confluence, the complete culture medium was replaced with a specific differentiation medium that contained 90% StemPro Osteocyte/Chondrocyte Differentiation Basal Medium (Invitrogen Life Science Technologies), 10% StemPro Chondrogenesis Supplement (Invitrogen Life Science Technologies), 100 UI/ml penicillin, 100 μg/ml streptomycin and 3 μg/ml amphotericin B (Invitrogen Life Science Technologies). The differentiation medium was replaced every 3 days for a total of 16 days of differentiation. After this procedure, the cells were fixed in 4% paraformaldehyde, washed in PBS and stained in 2 ml of 1% Alcian Blue (Sigma-Aldrich) in 0.1 N the cells were washed three times in HCl (Sigma-Aldrich) 0.1 N. The cells were incubated for 30 minutes at room temperature, after which the dye was removed, the cells were washed three times in 0.1 N hydrogen chloride (Sigma) and the fields were observed under an inverted light microscope.

### Differentiation into the osteogenic lineage

When the cell culture reached 70% confluence, the complete culture medium was replaced with a specific differentiation medium that contained 90% StemPro Osteocyte/Chondrocyte Differentiation Basal Medium, 10% StemPro Chondrogenesis Supplement, 100 UI/ml penicillin, 100 μg/ml streptomycin and 3 μg/ml amphotericin B. The differentiation medium was replaced every 3 days for a total of 10 days of differentiation. After this procedure, the cells were fixed in ice-cold 70% ethanol, washed in distilled water and stained in 2 ml Alizarin Red (Invitrogen Life Science Technologies, Carlsbad, CA, USA). The cells were incubated for 30 minutes at room temperature, after which the dye was removed, the cells were washed four times in water and the fields were observed under an inverted light microscope.

### *In vitro* spontaneous MSC differentiation in the presence of the biomaterial

The induction of spontaneous cell differentiation in the presence of FS was assessed after 20 days of MSC growth in the presence of the biomaterial. The specific markers that were described above for osteogenic, chondrogenic and adipogenic cell lineages were used to assess cell differentiation.

### Fibrin sealant and MSC formulations

Eight microliters of FS were added into six-well culture plates along with 3.20 × 10^5^ rat MSCs that had been cultured and characterized as described previously, followed by the addition of complete culture medium. The plates were kept in an incubator at 37.5°C in a humid atmosphere with 5% CO_2_. The maintenance medium was replaced every 5 days.

### Inverted light microscopy

The growth and interaction of MSCs (2.13 × 10^3^ cells/ml) with the biomaterial were evaluated in the first passage culture plates by inverted light microscopy.

### Fluorescence microscopy

Cells that grew on the biomaterial were evaluated for growth and viability after labeling with fluorescent probes. The cells were stained with Hoescht 33342 and propidium iodide (Sigma-Aldrich) to label live cells in blue and dead cells in red, respectively.

The medium and FS were removed from the MSC primary cell culture plate, and 0.5 ml PBS and 20 μl of 10 mg/ml Hoescht 33342 solution were added to the plate for 10 minutes, after which the PBS and Hoescht solution were discarded and the cells were washed with fresh PBS. Five μl of a 1 mg/ml propidium iodide solution were added to the plate and incubated for 5 minutes, after which the cells were washed again with PBS before analyzing the labeled cells with a fluorescence microscope (450 to 490 nm).

### Scanning electron microscopy

Sealant samples were cultured together with MSCs and were subsequently fixed in Karnovysky’s fixative (1% paraformaldehyde, 2.5% glutaraldehyde in 0.1 M phosphate buffer solution, pH 7.3) for 24 hours.

After the fixation step, the samples were washed with 0.1 M phosphate buffer, pH 7.3 (3× 5-minute washes), subjected to post-fixation in 1% osmium tetroxide diluted in the same buffer for 3 hours, dehydrated in increasing concentrations of ethanol up to a final concentration of 100%, transferred from a 70% concentration directly into absolute ethanol (this was repeated 3 times), dried in a CPD030 critical point apparatus with liquid carbon dioxide, mounted on appropriate stubs and, finally, metallized and gold-coated in a Sputter SCD050 (Leica Microsystems Inc. Buffalo Grove, IL. USA).

The analyses were performed with a JEOL Scanning Electron Microscope (JSM5800 LV; JEOL. Tokyo, Japan) at a voltage of 10 kV and were recorded as digital photographs by the same apparatus.

### Transmission electron microscopy

Sealant samples were cultured with MSCs and fixed in Karnovysky’s fixative (1% paraformaldehyde, 2.5% glutaraldehyde in 0.1 M phosphate buffer solution, pH 7.3) for 24 hours.

After fixation, the samples were washed with 0.1 M phosphate buffer, pH 7.3 (3× 5-minute washes), subjected to post-fixation in 1% osmium tetroxide diluted in the same buffer for 3 hours, dehydrated in increasing concentrations of ethanol up to a final concentration of 100%, immersed in acetone for 1 hour, immersed in acetone/resin (2:1) for 12 hours, immersed in acetone/resin (1:1) for 12 hours and embedded in Durcupan® ACM Fluka resin (Sigma-Aldrich). Embedded samples were polymerized in an oven at 50°C for 48 hours. Sections were cut with appropriate blades and stained as follows: 0.3 μm semi-thin sections were cut with a glass knife and stained with 1% Toluidine Blue on a hotplate, and 50 nm ultra-thin sections were cut with a diamond knife and stained with Manila/lead citrate (SPI Supplies. West Chester, PA. USA).

The analyses were performed with a TECNAI BIOTWEEN G2 SPIRIT Transmission Electron Microscope (FEI Company, Hillsboro, OR. USA) at a voltage of 80 kV and were recorded as digital photographs by the same apparatus.

## Results

### Fibrin sealant

The material formed a dense fibrin network that resulted in a stable clot approximately 2 minutes after the reconstitution of its components at room temperature.

### Mesenchymal stem cell expansion and characterization

**Figure 1:**
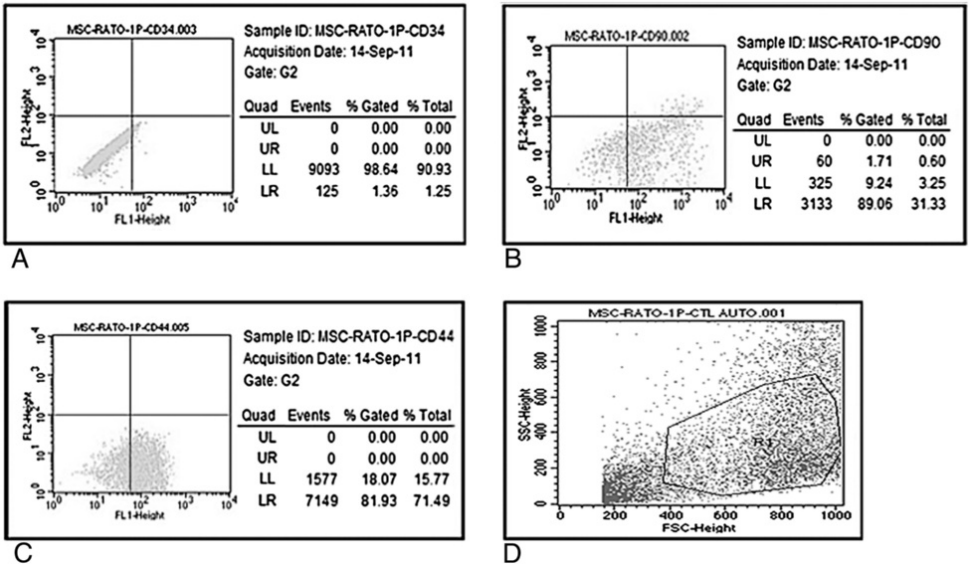
**Flow cytometric characterization of mesenchymal stem cells with negative and positive markers.** Flow cytometric characterization of mesenchymal stem cells (MSCs) with **(A)** negative (CD34) and **(B, C)** positive (CD90 and CD44) markers. **(D)** Cell population selected for analyses. FSC, forward scatter; LL, lower left; LR, lower right; SSC, side scatter; UL, upper left; UR, upper right.

MSCs from the primary culture were able to adhere to plastic, as confirmed by flow cytometry after the first passage. The flow cytometry results indicated that 1.36% of the cells expressed CD34 and 89.06% and 81.93% expressed CD90 and CD44, respectively, as shown in Figure 1A,B,C. Figure 1D shows the cell population selected for analyses.

No difference was observed in the cell growth rate after 12 days and 90% confluence regardless of the presence of FS. The average number of MSCs isolated in the first passage was approximately 6.41 × 10^5^.

### Differentiation into the adipogenic lineage

**Figure 2:**
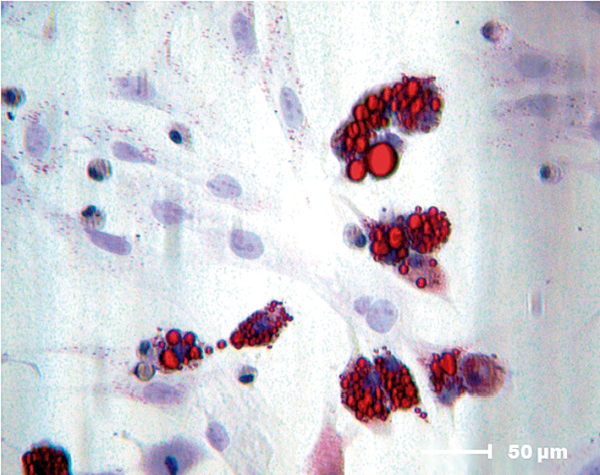
**Mesenchymal stem cell adipogenic differentiation potential.** Adipogenic differentiation (Oil Red-O (Invitrogen Life Science Technologies, Carlsbad, CA, USA); 400× magnification).

Lipid-rich vacuoles formed within the first-passage MSCs after 7 days of culture in specific differentiation media. These changes were noted by the presence of red-colored vacuoles that confirmed the adipogenic lineage (Figure 2).

There was no significant labeling of intracellular lipids in MSCs after 7 days of culture in the presence of FS, which indicated that the biomaterial alone could not induce the differentiation of MSCs to the adipogenic lineage.

### Differentiation into the chondrogenic lineage

**Figure 3:**
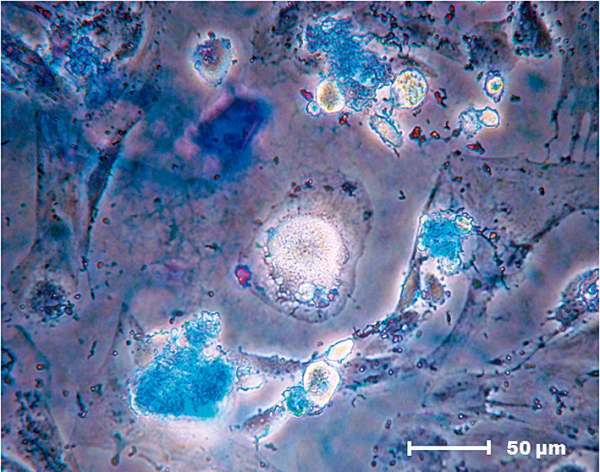
**Mesenchymal stem cell chondrogenic differentiation potential.** Chondrogenic differentiation (Alcian Blue (Sigma-Aldrich, St Louis, MO, USA); 400× magnification).

Proteoglycans produced by first-passage MSC chondrocytes were observed after 16 days of culture in specific differentiation media. These changes were noted by the presence of blue staining that confirmed the chondrogenic lineage (Figure 3).

There was no significant chondrogenic cell labeling after 16 days of MSC culture in the presence of FS, indicating that the biomaterial alone could not induce the differentiation of MSCs to the chondrogenic lineage.

### Differentiation into the osteogenic lineage

**Figure 4:**
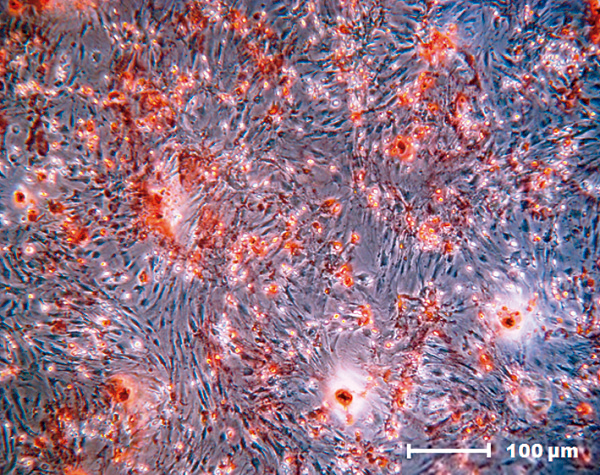
**Mesenchymal stem cell osteogenic differentiation potential.** Osteogenic differentiation (Alizarin Red (Invitrogen Life Science Technologies, Carlsbad, CA, USA); 200× magnification).

Calcium deposits were found in the MSC cultures after 10 days in specific differentiation media. The deposits were detected by the presence of red staining that labeled the mineral deposits in the extracellular medium and thus confirmed differentiation to the osteogenic lineage (Figure 4).

Low-intensity but significant staining was observed after 10 days of MSC culture in the presence of FS. Three additional 20-day cultures were performed. No labeling was observed in the MSC cultures in the presence or absence of the biomaterial prior to reaching maximum confluence. However, mineralization was observed in MSC cultures that were maintained for a long period at maximum confluence in the absence of FS. Cell labeling thus confirmed MSC differentiation to the osteogenic lineage in the absence of the biomaterial.

### Evaluation by inverted light microscopy

**Figure 5:**
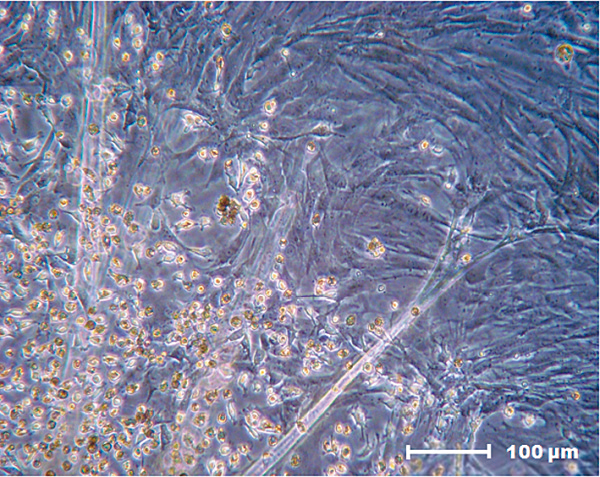
**Fibrin network that promotes mesenchymal stem cell capture.** Fibrin network with a cobweb appearance that promotes mesenchymal stem cell capture (200× magnification).

MSCs were mixed with FS after the first passage from the primary culture. The proliferation of cells with fibroblastoid morphology, including those around the biomaterial, was noted on the second day of culture. Cell confluence during this period was approximately 30 to 40%.The cells proliferated while maintaining their morphological characteristics and definition between the FS and MSCs after 4 days of culture. Maximum cell confluence in the cultures was 60 to 70%. The fibrin network formed by the FS displayed a cobweb appearance in some regions due to the presence of cells captured by the fibrin network (Figure 5).

The cell cultures were 90% confluent after 8 days in culture, and some of the MSCs adhered to and grew on the FS surfaces.

### Evaluation by fluorescence microscopy

**Figure 6:**
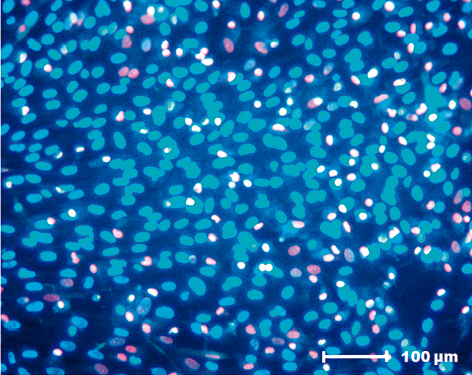
**Cell viability analysis of mesenchymal stem cells cultured in the presence of fibrin sealant.** Hoescht 33342 and propidium iodine (Sigma-Aldrich, St Louis, MO, USA) staining for the cell viability analysis of mesenchymal stem cells cultured in the presence of fibrin sealant. (200× magnification).

Viable cells were indicated by bright blue Hoescht 33342-stained nuclei. Cells with damaged membranes were stained red by propidium iodide. MSC viability was determined to be 80 to 85% in the presence of FS (Figure 6).

### Evaluation by scanning electron microscopy

**Figure 7:**
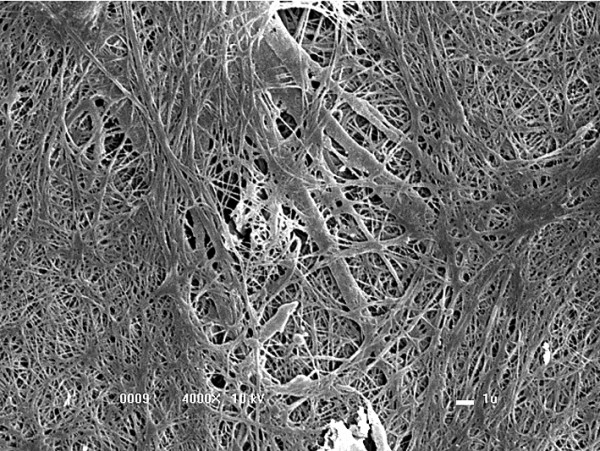
**Dense fibrin network formed by the fibrin sealant.** (4,000× magnification).

**Figure 8:**
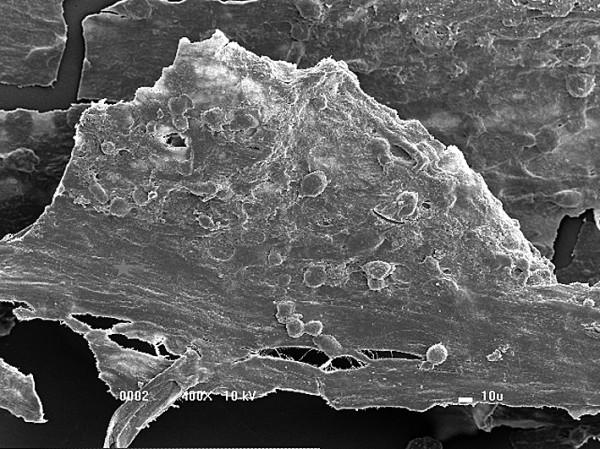
**Fibrin sealant and mesenchymal stem cells adhered to the surface of the biomaterial.** Mat formed by the fibrin sealant and the presence of a large amount of mesenchymal stem cells adhered to the surface of the biomaterial (400× magnification).

**Figure 9:**
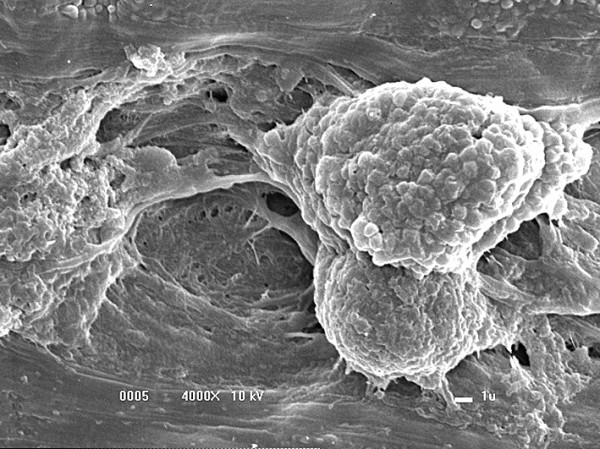
**Cellular prolongations towards the interior of the fibrin sealant.** (4,000× magnification).

The three-dimensional scanning electron microscopy analysis of FS alone revealed a uniform surface in which FS had formed a mat that primarily consisted of a dense fibrin network (Figure 7).Scanning electron microscopy analysis of the biomaterial after 8 days of MSC culture indicated the ability of FS to capture and promote cell adhesion on its surface (Figure 8). Interactions between adjacent cells and the presence of cellular extensions towards the interior of the biomaterial were observed at a higher magnification (Figure 9).

### Evaluation by transmission electron microscopy

**Figure 10:**
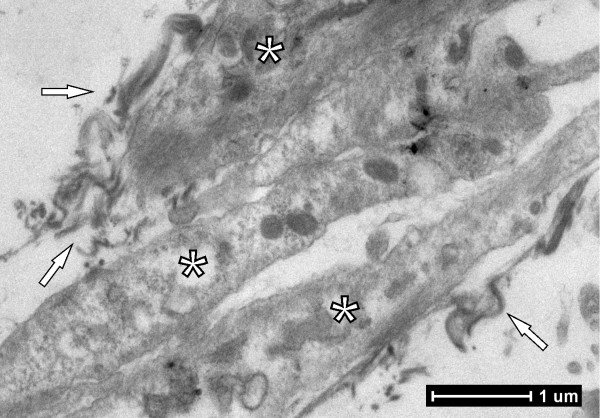
**Cell nucleus and deposited matrix formed by fibrin sealant.** Cellular projections with the presence of the cell nucleus (stars) and deposited matrix formed by fibrin sealant (arrows) (3,000**×** magnification).

The presence of cellular projections in regions where numerous layers had formed was observed during the analysis of images of the biomaterial acquired after 8 days of MSC culture on FS. The presence of cell nuclei, deposited matrix, possible original fibrin matrix and circular electrodense structures, which were characterized as mitochondria, were also observed. The images showed intimate connections between the MSCs and the scaffold, and thus it was possible to show contacts between the biomaterial interfaces and the MSCs (Figure 10).

## Discussion

MSCs are widely used because the cellular phenotype remains undifferentiated and the differentiation potential is maintained during long-term *in vitro* cultivation [6]. According to Bydlowski and colleagues, MSCs can differentiate into osteoblasts, chondrocytes and adipocytes both *in vivo* and *in vitro*[24]. This observation agrees with the results shown in this study, in which MSCs derived from rat bone marrow differentiated into the osteogenic, adipogenic and chondrogenic lineages.

Fu and colleagues showed that bone marrow MSC-derived neural stem cells expressed the characteristic MSC antigens CD29, CD44, CD73, CD90, CD105 and CD166 [25]. Furthermore, the MSCs could proliferate and differentiate into multiple mesodermal lineages. Mehlhorn and colleagues used fluorescein-conjugated CD34, CD45, CD90 and CD105 antibodies to characterize MSCs [26]. In the present study, MSCs of rat origin expressed high levels of CD44 and CD90 and low levels of CD34 as determined by flow cytometry. These findings are in agreement with the results reported by Fu and colleagues [25] and by Mehlhorn and colleagues [26].

According to Ahmed and colleagues, the most commonly used fibrin derivatives in tissue engineering are hydrogels, sealants and fibrin microbeads [3]. The new biomaterial FS, derived from snake venom, was first used as a biological scaffold and was shown to have optimal plasticity and an excellent ability to interact with MSCs. The ability of FS to induce the spontaneous differentiation of MSCs into the adipogenic and chondrogenic lineages was thus evaluated. No intracellular lipid deposit formation by adipocytes or proteoglycan production by chondrocytes was observed, indicating that the sealant did not directly affect MSC differentiation.

The onset of spontaneous differentiation to the osteogenic lineage in the presence of FS was as determined by significant Alizarin Red staining of osteoblasts. When a similar culture was prepared in low-glucose complete medium without FS and allowed to reach maximum confluence during a 20-day period, MSCs also differentiated into osteogenic lineage cells. These results confirm that the MSCs were driven to differentiate by a long culture period at maximum confluence rather than by the presence of the biomaterial. The undifferentiated MSCs might also have been stimulated to differentiate to the osteogenic lineage as a result of the collection site or the presence of bone cell contaminants. The inability of FS to induce differentiation and the neutrality of FS in MSC culture were therefore again demonstrated.

The interaction of cells with the FS surface, which was observed by optical microscopy and scanning electron microscopy, showed large amounts of MSC adherence and growth on the dense fibrin network formed by the sealant. Gardin and colleagues also noted this phenomenon and reported interactions between adipose tissue-derived stem cells and scaffolds produced from traditionally prepared FS and hyaluronic acid [27].

Transmission electron microscopy showed the presence of nuclei with euchromatic features, which highlighted active protein synthesis along with high metabolic activity. We report the existence of interactions between viable and actively functional MSCs and the FS, due to the clear presence of deposited matrix. These results are corroborated by Trubiani and colleagues, who reported extensive MSC biomass growth on scaffolds, including a fibrin sponge [28].

A cell viability test with markers for live (Hoescht 33342) and dead (propidium iodide) cells indicated viability rates above 80% for MSCs cultured in the presence of FS. Such a result ensures the presence of excellent interactions between the cells and the scaffold (FS) because the cells were able to maintain their viability and functionality. This result corroborates an earlier report by Yamada and colleagues that showed the possibility of transferring MSCs to receptor sites without losses in cellular structure integrity or viability of the cultured tissue [29]. Yamada and colleagues also showed that stem cells could effectively migrate and interact on different scaffolds derived from traditionally prepared fibrin sealants [29].

Several *in vivo* studies have been performed in different animal species and humans, demonstrating the translational potential of our FS [16,30,31].

## Conclusions

The snake venom-derived FS produced at the Center for the Study of Venoms and Venomous Animals at UNESP interacts very well with rat bone marrow-derived MSCs. Moreover, this new FS can be prepared and adjusted according to MSC needs, thus enabling use with autologous, homologous or heterologous fibrinogen. Based on the results, this new FS is a promising biological three-dimensional scaffold candidate – and because the product is derived from animals, FS does not transmit infectious diseases from human blood.

## Abbreviations

FS: fibrin sealant; MSC: mesenchymal stem cell; PBS: phosphate-buffered saline.

## Competing interests

This research is under the scope of the Brazilian Patents BR 10 2014 011432 7 and BR 10 2014 011436 0.

## Authors’ contributions

VPOG made substantial contributions to conception and design, acquisition, analysis and interpretation of data. FCL-A made substantial contributions to conception and design. ALRO made substantial contributions to acquisition, analysis and interpretation of data. GFS made substantial contributions to acquisition, analysis and interpretation of data. JFL-N made substantial contributions to acquisition, analysis and interpretation of data. BB was involved in drafting the manuscript and revising it critically for important intellectual content. RSFJr was involved in drafting the manuscript and revising it critically for important intellectual content, and made substantial contributions to conception, design, analysis and interpretation of data. All authors read and approved the final manuscript.
